# Brushing behavior among young adolescents: does perceived severity matter

**DOI:** 10.1186/1471-2458-14-8

**Published:** 2014-01-08

**Authors:** Parisa Kasmaei, Farkhondeh Amin Shokravi, Alireza Hidarnia, Ebrahim Hajizadeh, Zahra Atrkar-Roushan, Kambiz Karimzadeh Shirazi, Ali Montazeri

**Affiliations:** 1 Department of Health Education, Faculty of Medical Sciences, Tarbiat Modares University, Tehran, Iran; 2 Department of Biostatistics, Faculty of Medical Sciences, Tarbiat Modares University, Tehran, Iran; 3 Department of Health Education, Faculty of Medical Sciences, Yasuj University, of Medical Sciences, Yasuj, Iran; 4 Mental Health Research Group, Health Metrics Research Center, Iranian Institute for Health Sciences Research, ACECR, Tehran, Iran

## Abstract

**Background:**

Oral health is a basis for general health and well-being and affects physical and psychological aspects of the human life. The aim of this study was to determine the power of the health belief model in general and the role of perceived severity and its components in particular in predicting tooth brushing behavior among young adolescents.

**Methods:**

This was a cross sectional study of a sample of female students grade four in Rasht (a metropolitan in north Iran) in 2012. A systematic random sampling method was applied to recruit students. They were asked to respond to a designed questionnaire containing items on brushing behavior based on the health belief model. In this study for the first time perceived severity and perceived barriers were divided into two parts, perceived subjective and objective severity and perceived physical and psychological barriers and were treated as independent variables. Logistic regression analysis was performed in order to identify the variables that predict the desirable behavior (brushing twice a day or more).

**Results:**

In all 265 female students were entered into the study. Of these, only 17.4% reported that they were brushing at least twice a day (desirable behavior). The results obtained from the logistic regression analysis indicated that perceived objective severity (OR = 0.37, 95% CI = 0.21- 0.66, P = 0.001) and feeling less perceived psychological barriers (OR = 2.60, 95% CI = 1.50- 4.52, P = 0.001) were the significant predicting factors for brushing twice a day.

**Conclusion:**

The findings suggest that perceived objective severity and perceived psychological barriers play important role in adapting a desirable health behavior among young adolescents.

## Background

Oral health is fundamental to general health and well-being [1] and affects physical and psychological aspects of the human life [2]. A desirable oral health is crucial for quality of life, self-esteem and social confidence. It helps individuals communicate effectively, speak, eat, enjoy food and prevent diseases, discomfort or embarrassment [2,3]. Thus it is argued that oral health should be seriously considered from childhood. Unfortunately, the prevalence of dental problems among children is high [4]. Dental caries affects 60-90% of school-aged children. The distribution and severity of dental caries vary in different parts of the world as well as within the same region or country [5].

According to the Iranian Ministry of Health, most Iranians do not adequately pay attention to oral health. Since this is the same for high-income families, it seems that this problem is rather a cultural habit and does not solely relate to people’s economic situations (personal communication). Thus, if one wishes to implement an intervention to change people’s behavior, it should be noted that in such processes we usually face what people believe on their subjective rather than the objective world of a physician or a physicist [6,7]. As such designing interventions based on the Health Belief Model might be more relevant.

The Health Belief Model (HBM) is one of the first theories concerned with the health-related behaviors. It provides specific guidance at micro level for planning interventions for behavior change and now it is one of the most popular models in health education and promotion [8]. Because of its dominant emphasis on prevention, it usually is applied in health protection activities while other theories such as health promotion models are more action-oriented [9].

The Health Belief Model is used to predict and explain behavioral changes in dental health [10] and contains five main constructs: perceived susceptibility, perceived severity, perceived benefits, perceived barriers, and self-efficacy. These were defined as:

“1. Perceived susceptibility: Subjective belief that a person may acquire a disease or enter a harmful state as a result of a particular behavior.

2. Perceived severity: Belief in the extent of harm that can result from the acquired disease or harmful state as a result of a particular behavior

3. Perceived benefits: Belief in the advantages of the methods suggested for reducing the risk of seriousness of the disease or harmful state resulting from a particular behavior.

4. Perceived barriers: Belief concerning actual and imagined costs of performing the suggested behavior.

*5. Self-efficacy: Confidence in one’s ability to acquire a new behavior*[8]*.”*

Most investigators suggest that from the above mentioned constructs; perceived barriers and self-efficacy are the most important constructs that could predict a health behavior [11-15]. Yet, fewer investigators suggest that perceived barriers is not a significant construct in predicting health behaviors [16,17]. However, there is evidence that perceived severity of oral diseases is associated with increased tooth brushing frequency or oral health behavior [13,16-18]. The combination of perceived severity and perceived susceptibility is usually named as perceived threat. It has a great relevance to many health behaviors [19]. Thus we were interested to investigate more on this construct in order to contribute to the literature on the topic. In particular we thought to examine whether perceived severity is associated with tooth brushing behavior among young adolescents, and if this is the case then to investigate about its components to better understand the issue.

As indicated the theoretical framework for this study was built on the bases of the Health Belief Model where it has been suggested that attempting to influence the regular tooth brushing (twice a day or more) requires an understanding of the beliefs that strengthen this behavior [18]. According to the Health Belief Model, previous investigators reported contradictory findings. A group of investigators have found that the model was good in predicting oral health behavior (13,17), while the others reported that although the model could be used, its limitations should be recognized (11,16). Thus, we aimed to investigate the model to see how this works in preadolescents. In fact our first hypothesis was that oral health beliefs would have considerable predicting power in tooth brushing frequency in preadolescents. The second hypothesis was to find out which constructs of the Health Belief Model could predict tooth-brushing frequency in preadolescents.

## Methods

### Design and data collection

This was a cross sectional study conducted on 9–10 years old female students (grade four) in Rasht (a metropolitan in north Iran) in 2012. For the data collection a list of all governmental female schools were provided (66 schools) and a systematic random sampling was applied (one out of 3 schools) in order to insure that every region in the city represent in the study. The estimated number of students sampled from each school was based on the fact that we thought for factor analysis at least a sample of 250 students is needed (50 students per each main construct of the Health Belief Model). Usually there were 20 to 25 grade four students within each school. Then, within each school 12 students were recruited by a simple random sampling approach (with one exception where 13 students were recruited). In each school students were asked to respond to the study questionnaire. They were free to reject the invitation. Fortunately all students accepted the invitation and participated in the study. The main investigator was present to help students while they were responding to the questionnaire. Due to regulations since the main investigator was female she was not allowed to attend to the boys’ schools.

### The questionnaire

The questionnaire was consisted of 8 items related to demographic information, 26 items related to the health belief model constructs including: perceived susceptibility, perceived severity, perceived benefits, perceived barriers, perceived self efficacy, and 3 items on reasons for visiting dentist, sources of information, and brushing behavior frequency. The items derived from previous studies on the topic [11,13,14,20]. A panel of experts consisting of ten faculty members (eight specialized in health education and two dentists) evaluated the content validity. Consequently psychometric properties of the questionnaire were examined. The construct validity was assessed by performing explanatory factor analysis and reliability was evaluated by assessing internal consistency. The results obtained from explanatory factor analysis with oblique rotation, indicated a 7-factor solution that jointly accounted for 59.6% of variance observed. We noticed that ‘perceived severity’ was loaded on two separate factors (perceived subjective and objective severity) and similarly ‘perceived barriers’ also was loaded on two parts (perceived physical and perceived psychological barriers). The constructs and sample items in addition to the Cronbach’s alpha coefficients are shown in Table 1 (the detailed results are not shown but is available from the main investigator).

**Table 1:** Constructs and sample items

**Constructs (number of items)**	**Sample item**	**Possible score range**	Cronbach’s alpha coefficient*
Perceived susceptibility (3)	Only when I am young my teeth would be healthy.	3-15	0.77
Perceived subjective severity (3)	Tooth decay will cause me bad breath smell.	3-15	0.76
Perceived objective severity (4)	Tooth decay will make me look bad when I am laughing.	4-20	0.81
Perceived benefits (3)	Brushing my teeth can prevent tooth decay.	3-15	0.71
Perceived psychological barriers (5)	When I am tired I do not brush my teeth.	5-25	0.85
Perceived physical barriers (2)	My gums will bleed when I brush my teeth.	2-10	0.76
Self-efficacy (5)	I am confident that I can brush my teeth even when I am tired.	5-25	0.86

*Values equal or grate than 0.7 are considered satisfactory.

### Statistical analysis

Descriptive statistics used to explore the data. Then, comparison was made between those who were brushing less than twice a day and those who were brushing twice or more per day using Mann–Whitney U test. Finally logistic regression analysis was performed in order to assess the association between dependent variable (brushing behavior) and independent variables including perceived susceptibility, perceived subjective severity, perceived objective severity, perceived physical barriers, perceived psychological barriers, and perceived self-efficacy.

### Ethics

Ethic committee of Tarbiat Modares University approved the study. Consent was obtained from parents and authorities.

## Results

In all 265 female students (9–10 years old primary school, grade 4) were participated in the study. Overall 17.4% of the students (n = 46) reported that they were brushing twice a day or more (group 1), while the remaining 219 students (82.6%) reported that they were brushing less than twice a day (group 2). The main reason for visiting the dentist was decay/pain/break (77%). The most frequently reported sources of information were health personnel (75.3%) and parents (57.5%). The findings are presented in Table 2.

**Table 2:** The frequency of brushing behavior, reason for visiting dentist and sources of information

	**Number**	**Percentage**
**Brushing behavior**		
Brushing less than twice a day	219	82.6
Brushing twice a day or more	46	17.4
**Reason for visiting dentist**		
Decay/Pain/Break	204	77
Annual check up	36	13.6
Six months check	25	9.4
Sources of information*		
Health personnel	55	75.3
Parents	42	57.5
Teachers lecture	22	30.1
Television	16	21.9
Role playing in the class	13	17.8
Brochure	6	8.2
Others (radio, peers, sister, pharmacy)	8	11.1
None	6	8.2

*Respondents could identify more than one source.

Table 3 summarizes the students’ scores for different constructs. There were significant differences between those who were brushing twice a day or more and those who were brushing less than twice a day on several measures including perceived objective severity (P = 0.001), perceived psychological barriers (P < 0.0001), and perceived self-efficacy (P < 0.0001). However, the other constructs did not show any significant results.

**Table 3:** Comparison of the health belief model constructs among students with and without desirable brushing behavior*

	**All (n = 265)**	**Brushing less than twice a day (n = 219)**	**Brushing twice a day or more (n = 46)**	
	**Mean (SD)**	**Mean (SD)**	**Mean (SD)**	**P**
Perceived susceptibility	9.4 (3.11)	8.5 (3.07)	8.7 (3.32)	0.65
Perceived subjective severity	12.1 (2.81)	12.0 (2.84)	12.6 (2.62)	0.25
Perceived objective severity	14.3 (3.17)	14.0 (3.21)	15.8 (2.56)	<0.0001
Perceived benefits	13.0 (2.66)	12.9 (2.71)	13.6 (2.33)	0.11
Perceived psychological barriers	13.6 (5.37)	14.2 (5.40)	10.7 (4.22)	<0.0001
Perceived physical barriers	6.0 (2.54)	6.12 (2.53)	5.71 (2.59)	0.34
Self-efficacy	17.9 (4.71)	17.4 (4.61)	20.1 (4.67)	<0.0001

*Higher scores for perceived susceptibility, perceived subjective severity, perceived objective severity, perceived benefit and self-efficacy indicate better conditions. For perceived psychological barriers and perceived physical barriers lower scores indicate better conditions.

In order to determine the association between the desirable behavior (brushing twice a day or more), and independent variables including perceived susceptibility, perceived subjective severity, perceived objective severity, perceived physical barriers, perceived psychological barriers, and perceived self-efficacy logistic regression analysis was performed. In doing so the influential cases and outliers, based on Cook’s distances and standardized residuals was applied. Excluding outliers, 258 students entered in the analysis and the full model significantly was reliable (k^2^ = 42.6, df =7, P < 0.0001). This model explained 25% to 37% of variance for observed undesirable behavior (Cox = 0.25; Snell R Square = 0.37). The classification accuracy rate obtained from logistic regression analysis was 98.2% for undesirable behavior and 27.9% for desirable behavior. However, the results indicated that perceived objective severity had a preventive effect on the risk of brushing less than twice a day (OR = 0.37, 95% CI = 0.21- 0.66, P = 0.001) and feeling higher perceived psychological barriers increased the risk of not performing the desirable behavior (OR = 2.60, 95% CI = 1.50- 4.52, P = 0.001). The results are shown in Table 4.

**Table 4:** Factors predicting undesirable brushing behavior among students

	**B**	**SE**	**Wald statistics**	**OR (95% CI)**	**P**
Perceived susceptibility	-0.18	0.19	0.87	1.19 (0.83- 1.72)	0.35
Perceived subjective Severity	-0.07	0.22	0.11	0.93 (0.60- 1.44)	0.74
Perceived objective Severity	-0.99	0.29	11.42	0.37 (0.21- 0.66)	0.001
Perceived benefits	-0.11	0.26	0.17	0.90 (0.54-1.50)	0.68
Perceived psychological barriers	0.96	0.28	11.49	2.60 (1.50- 4.52)	0.001
Perceived physical barriers	0.04	0.16	0.06	0.96 (0.70- 1.32)	0.81
Self-efficacy	-0.13	0.30	0.20	0.88 (0.49- 1.57)	0.66

## Discussion

The findings from this study indicated that perceived objective severity and perceived psychological barriers were the most important factors in predicating tooth brushing behavior in young adolescents. In this study more than 80% of the students claimed that they do not brush twice a day. These findings suggest that there is urgent need for planning and performing appropriate and rapid educational programs.

We found that one unit increase in perceived objective severity increased the possibility of desirable behavior about three times. The finding showed that this construct had a strong power in predicting tooth-brushing behavior. In general perceived severity is a feeling that might help an individual to perform a given health behavior. However, for the first time we found that perceived severity contained two distinct concepts namely objective severity and subjective severity. In fact this classification indicated that it is the perceived objective severity that tiger performing a desirable behavior and not the perceived subjective severity. Apparently, this classification led us to formulate an argument that since this age group of students did not have abstract thinking and can only have objective imagination thus firstly perceived severity could be two types (objective and subjective) and secondly this is the objective severity that plays a role and not subjective severity when a target group is a young adolescence. Interestingly we found that Piaget’s Theory of Cognitive Development supports this argument and identifies that children during the concrete operational stage that lasts from age 7 to about age 12, can deal with concrete objects rather than with abstractions when they consider change. They must either see or be able to imagine objects. They have difficulty with abstract terms and cannot reason in a scientific, and deductive manner [21]. In the current study bad breath smell is an example of abstractions that named ‘perceived subjective severity’ and ‘missing the ability of good speaking or eating’ are examples of concrete objects that named ‘perceived objective severity’.

In general there are controversial findings on predictive power of the perceived severity. A number of studies reported favorable results [13,16-18] while some did not support that perceived severity could predict a behavior change [11,12]. In any case it should be noted that perceived severity has a well-built cognitive component, which is dependent on one’s knowledge. Therefore, it is argued that in designing health education programs in addition to personalizing, there is need to include perceived severity by describing the serious negative consequences of not complying with a recommendation [8]. In other words a person must feel threatened by his/her current behaviors (perceived susceptibility and perceived severity) and believe that the outcome of changing his/her behavior is valuable and will lead to an acceptable benefit. Thus it has been suggested that before perceived susceptibility becomes a powerful predictor it is necessary to achieve to a heightened situation of severity [19].

According to a novel finding from this study where we identified two types of perceived barriers (physical and psychological), there was a meaningful reverse relationship between the psychological section and the desirable behavior but not between the perceived physical barriers and the desirable behavior. One score reduction in the perceived psychological barriers increased the possibility of desirable behavior more than two times. The relationship between the perceived barriers (without classification) and the desirable behavior change has been confirmed in various studies [11-15,18], although some studies did not support this finding [16,17]. As noted by several investigators, the perceived barriers are the most important predictors of behaviors in the HBM [8]. Therefore, one might argue that reducing barriers could be one of the best strategies to encourage oral self-care [11].

We found that perceived self-efficacy was significantly associated with proper tooth brushing behavior in univariate analysis but contrary to findings from other investigations (11,14,15,16) it did not emerge as a significant factor in logistic regression analysis. Perhaps such an observation confirms our previous argument indicating that children from age 7 to about age 12 can deal with concrete objects rather than with abstractions. In fact one might argue that perceived self-efficacy is a process of abstract thinking rather than an immediate concrete action to perform a behavior. Then one might question why the current study did measure ‘subjective severity’ in children with no ability to think in abstract terms? And why these children can think about ‘psychological barriers’? However, with regard to the study results, it seems that perceived objective severity and perceived psychological barriers may play a key role when designing educational interventions.

### Limitations

As most participants in this study were female students in a metropolitan city, cautious in interpretation is needed in generalizing the findings to male students or other populations. Even, further studies with similar age groups are necessary for confirming the findings.

## Conclusion

The findings suggest that perceived objective severity and perceived psychological barriers play important role in adapting a desirable health behavior among young adolescents.

## Competing interests

The authors declare that they have no competing interests.

## Authors’ contributions

PK was the main investigator, collected the data, designed the study and wrote the first draft. FAS supervised the study. AH, EH, ZAR, KKS were the study consultants. AM critically reviewed and edited the manuscript, responded to the reviewers comments and provided the final draft. All authors read and approved the manuscript.

## Pre-publication history

The pre-publication history for this paper can be accessed here:

http://www.biomedcentral.com/1471-2458/14/8/prepub
